# Editorial to the Research Topic “Comparative studies between HTLV-1 and HTLV-2 function and pathobiology”

**DOI:** 10.3389/fmicb.2014.00792

**Published:** 2015-01-14

**Authors:** Umberto Bertazzoni

**Affiliations:** Department of Life and Reproduction Sciences, Section of Biology and Genetics, University of VeronaVerona, Italy

**Keywords:** HTLV-1, HTLV-2, expression, proteins, co-infection

Human T-cell leukemia viruses type 1 and 2 (HTLV-1 and HTLV-2) share a common genetic organization, expression strategy and ability to infect and immortalize T-cells *in vitro*; however, HTLV-1 and HTLV-2 are strikingly different in terms of clinical impact. HTLV-1 is recognized as the aetiological agent of adult T-cell leukemia/lymphoma (ATLL), and HTLV-associated myeolopathy/tropical spastic paraparesis (HAM/TSP), in contrast, HTLV-2 does not cause hematologic disorders and is only sporadically associated with cases of subacute myelopathy. HTLV-1 and HTLV-2 also exhibit distinct cellular tropisms *in vivo*: HTLV-1 is mainly found in CD4+T lymphocytes, whereas CD8+T-cells are the preferred target for HTLV-2.

The articles contributed in this Research Topic are covering all the different aspects that characterize HTLV-1 and HTLV-2, by highlighting differences in their biology that might provide clues to their distinct pathogenic properties.

Emphasis was placed on the comparison of mRNA expression (Cavallari et al., 2013), the genetic organization and expression patterns (Ciminale et al., 2014), the structural and functional properties of HTLV-1 and HTLV-2 Tax proteins (Forlani et al., 2013; Ren and Cheng, 2013; Romanelli et al., 2013; Shirinian et al., 2013), the role of accessory proteins and antisense proteins in viral pathogenesis (Anupam et al., 2013; Barbeau et al., 2013), and the mechanisms of HIV-1/HTLV-1 and HIV-1/HTLV-2 co-infections (Pilotti et al., 2013).

The fine tuning of expression of HTLV-1 and HTLV-2 (Cavallari et al., 2013) was focused on the X region that codes for the regulatory and accessory proteins in partially overlapping ORFs. Expression of such compact genomes is accomplished by a combination of ribosomal frameshifting, alternative splicing and polycistronic translation as well as production of negative-strand transcripts that code for the HBZ and APH-2 proteins. Recent studies of the temporal sequence of HTLV-1 gene expression in PBMCs isolated from infected patients revealed a “two-phase” kinetics in which tax/rex mRNA expression precedes that of other viral transcripts. A similar analysis of HTLV-2 mRNA expression indicated a comparable expression pattern, although HTLV-2 is characterized by a more abundant expression of gene products that may favor viral latency (i.e., p28 and tRex).

Given the relevance of Tax in the initial steps of T-cell transformation, their key role as the main viral oncoproteins was discussed in four different articles, each one focusing on a specific aspect of Tax function. Romanelli et al. (2013), highlighted distinctive properties of Tax proteins with emphasis on the activation of the NF-κB pathways and interactions with host factors that participate in signal trasduction. Tax-1 and Tax-2, though sharing many common properties, significantly differ for the presence of a PDZ motif, which is missing in Tax-2. This motif mediates the interaction of Tax with host factors regulating the cell cycle; in addition Tax-2 is unable to activate the non-canonical NF-κB which is attributed only to Tax-1. Shirinian et al. (2013), specifically addressed the subcellular localization and trafficking of Tax-1 and Tax-2, and their effects on cellular regulatory proteins. A special attention was given to Tax-1/Tax-2 post-translational modifications, as well to NF-κB activation and protein-protein interactions involved in oncogenecity both *in vivo* and *in vitro*. Ren and Cheng (2013), discussed their recent observations and views on the differential transforming activity of Tax-1 and Tax-2 in human T cells. Both proteins are unable to immortalize CD8 cells but Tax-2 is more efficient than Tax-1 in immortalizing CD4 cells. Forlani et al. (2013), discussed the pivotal role of the class II transactivator (CIITA) in triggering the adaptive immune response against pathogens. CIITA acts as an endogenous restriction factor against HTLV-1 and HTLV-2 by targeting their viral transactivators Tax-1 and Tax-2. The authors reviewed their findings on CIITA-mediated inhibition of viral replication and discussed similarities and differences in the molecular mechanisms by which CIITA specifically counteracts the function of Tax proteins.

The function of the Rex protein was presented by Ciminale et al. (2014). Although Rex-1 and Rex-2 share a similar domain structure, the truncated forms of Rex-2 are capable of inhibiting Rex function, while their HTLV-1 homolog p21 Rex might lack this activity.

The role in viral replication and viral pathogenesis of accessory proteins HTLV-1 p30 and HTLV-2 p28 were highlighted by Anupam et al. (2013). Both p30 and p28 regulate viral gene expression at the post-transcriptional level whereas p30 can also function at the transcriptional level. Since p30 and p28 have distinct interactome profiles even though they both interact with similar proteins, they must have divergent functions during the lifecycle of the viruses.

The functions of other accessory proteins have been presented by Ciminale et al. (2014). HTLV-1 p13 and p8 are not expressed by HTLV-2 which codes for p11, whose function is still unclear and does not seem to have a homolog in HTLV-1.

The possible role played by antisense proteins HTLV-1 HBZ and HTLV-2 APH-2 in the establishment of pathologies induced by viral infection has been carefully considered by Barbeau et al. (2013). Unlike APH-2, HBZ possesses specific domains which mediate its capacity to enhance its own expression, deregulate several pathways and induce T cell proliferation, providing substantial evidence toward its implication in viral persistence and ATLL cell survival.

Finally the problem of HIV/HTLV co-infection has been discussed by Pilotti et al. (2013). During co-infection with HIV-1, HTLV-2 modulates the cellular microenvironment favoring its own propagation and inhibiting HIV-1 progression. Possible differences between HTLV-1 and HTLV-2 on innate immune mechanisms induction and a particularly impact on NK cells are becoming evident.

In conclusion, in this Research Topic, HTLV-1 and HTLV-2 have been thoroughly compared and major differences outlined and discussed. Of particular interest is that HTLV-2 infection, contrary to HTLV-1, is characterized by an oligoclonal distribution in asymptomatic hosts; this indicates that the etiology of malignant transformation by HTLV-1 cannot be uniquely attributed to this phenomenon and further investigation is needed to understand why HTLV-2 is defective in promoting leukemogenesis.

## Conflict of interest statement

The author declares that the research was conducted in the absence of any commercial or financial relationships that could be construed as a potential conflict of interest.
